# TLR4 counteracts BVRA signaling in human leukocytes via differential regulation of AMPK, mTORC1 and mTORC2

**DOI:** 10.1038/s41598-019-43347-8

**Published:** 2019-05-07

**Authors:** Zhiyong Zhang, Louis F. Amorosa, Anna Petrova, Susette Coyle, Marie Macor, Mohan Nair, Leonard Y. Lee, Beatrice Haimovich

**Affiliations:** 1 Department of Surgery, Rutgers Robert Wood Johnson Medical School (RWJMS), New Brunswick, 08903 NJ USA; 2 Department of Medicine, Rutgers Robert Wood Johnson Medical School (RWJMS), New Brunswick, 08903 NJ USA; 3 Department of Pediatrics, Rutgers Robert Wood Johnson Medical School (RWJMS), New Brunswick, 08903 NJ USA

**Keywords:** Cell signalling, Neutrophils

## Abstract

TLR4 is implicated in diseases associated with chronic low-grade inflammation, yet homeostatic signaling mechanisms that prevent and/or are affected by chronic TLR4 activation are largely uncharacterized. We recently reported that LPS/TLR4 activates in human leukocytes signaling intermediates (SI), abbreviated TLR4-SI, which include mTORC1-specific effectors and targets, and that leukocytes of patients with T2D or after cardiopulmonary bypass (CPB) expressed similar SI. Extending these previous findings, here we show that TLR4-SI expression post-CPB was associated with low serum bilirubin and reduced preoperative expression of biliverdin reductase A (BVRA), the enzyme that converts biliverdin to bilirubin, in patient’s leukocytes. Biliverdin inhibited TLR4 signaling in leukocytes and triggered phosphorylation of mTORC2-specific targets, including Akt, PKCζ, AMPKα-LKB1-TSC1/2, and their association with BVRA. Torin, PP242, and a PKCζ inhibitory peptide, but not rapamycin, prevented these biliverdin-induced responses and TLR4 inhibition. In contrast, LPS/TLR4 triggered decreases in BVRA, AMPKα and PKCζ expression, and an increase in haptoglobin, a heme binding protein, in leukocytes *in vivo* and *in vitro*, indicating that activated TLR4 may suppress biliverdin/BVRA signaling. Significantly, compared to non-diabetics, BVRA and PKCζ expression was low and haptoglobin was high in T2D patients leukocytes. Sustained TLR4 activation may deregulate homeostatic anti-inflammatory BVRA/mTORC2 signaling and thereby contribute to chronic inflammatory diseases.

## Introduction

Toll-like receptor 4 (TLR4) is implicated in morbidities associated with chronic low-grade inflammation, including insulin resistance and type 2 diabetes (T2D)^1,2^. Our understanding of homeostatic molecular mechanisms that limit TLR4 signaling, and whether/how chronic TLR4 activation might impact these homeostatic signaling processes remains limited. TLR4 is activated by lipopolysaccharide (LPS) derived from pathogenic and commensal gut-residing Gram-negative bacteria, and endogenous ligands that include free fatty acids^3,4^. LPS is a potent inducer of inflammatory responses in humans^5,6^. However, whereas LPS doses above 0.5 ng/kg elicited inflammatory responses that included IL-6 and TNF-α production and broad changes in leukocyte’s mRNA and signaling intermediates (SI) expression^7^, doses below 0.5 ng/kg triggered only changes in leukocyte’s SI expression^8^. These observations suggested the hypothesis that the pattern of SI detected in leukocytes challenged with low LPS doses may be clinically relevant and serve as a tool for interrogating whether and how chronic low-grade TLR4 activation may deregulate homeostatic signaling mechanisms that prevent unwarranted TLR4 activation.

Among the SI affected by TLR4 activation are several mammalian target of rapamycin (mTOR) complex 1 (mTORC1) signaling components^9,10^, including AMP-activated protein kinase (AMPK) and hypoxia inducible factor subunit α (HIF-1α)^8^. In general, mTORC1 is activated when nutrients are abundant. Scarcity of energy and/or glucose triggers AMPK activation^11^. AMPK can prevent mTORC1 signaling via two mechanisms: (i) AMPK can phosphorylate Raptor on Ser792 thus preventing mTORC1 assembly^12^, and (ii) AMPK can phosphorylate and activate TSC1/2^13^, which acts as a GAP for Rheb^14^. Activated TSC1/2 inhibits Rheb and consequently mTORC1^14^. Raptor is phosphorylated at Ser792 in resting leukocytes^9,10^. LPS/TLR4 triggers Raptor dephosphorylation at Ser792 via a unique matrix metalloproteinase 9 (MMP9)-dependent molecular mechanism^9^. Though MMP9 is best known for its role in extracellular matrix degradation^15^, in LPS activated leukocytes intracellular MMP9 contributes to AMPKα cleavage^9,10^, mTORC1 activation, and the phosphorylation of S6K1 at Ser389^16,17^. mTORC1 also upregulates TLR4 and HIF-1α expression. HIF-1α and HIF-1β form the transcription factor HIF-1, an essential regulator of myeloid cells bioenergetics and antimicrobial functions^18^. LPS does not activate mTOR complex 2 (mTORC2)^10^. Hereafter, we refer to the panel of SI that include elevated MMP9, HIF-1α, TLR4, and proteolytically cleaved AMPKα, as ‘TLR4-SI’.

We recently reported that leukocytes of patients with T2D and a majority of patients after cardiopulmonary bypass (CPB) surgery expressed SI similar to TLR4-SI^9,10^. However, T2D patients treated with insulin, in general, did not express TLR4-SI^10^. Further investigation revealed that insulin counter-regulates TLR4 signaling in leukocytes, and *vice-a-versa*^10^. Building on this prior work, in this study we sought to determine why the majority, but not all CPB patients expressed TLR4-SI postoperatively, hypothesizing existence of TLR4 signaling regulator(s) in addition to insulin. Here, a prospective blinded study of CPB patients uncovered an inverse association between postoperative TLR4-SI expression and serum bilirubin levels. Bilirubin is the end product of heme catabolism. Heme is converted to biliverdin by heme oxygenases and then reduced to bilirubin by biliverdin reductase A (BVRA). We then discovered that TLR4-SI expression postoperatively was also inversely associated with preoperative BVRA expression, suggesting that TLR4- and BVRA-signaling in leukocytes are interconnected. We report that biliverdin/BVRA activates mTORC2 and an AMPK signaling axis that inhibits mTORC1, a key TLR4 signaling node. Furthermore, we identify biliverdin/BVRA SI that are altered by TLR4 causing mTORC2 inhibition. The data identify a novel counter-regulatory signaling loop involving BVRA and TLR4. We propose that in T2D patient’s leukocytes this loop is deregulated and that the signals are skewed in favor of TLR4.

## Materials and Methods

All methods were performed in accordance with the relevant guidelines and regulations.

### Study protocol, inclusion exclusion criteria, and surgical procedure

Rutgers, Robert Wood Johnson Medical School Institutional Review Board approved the study. Patient participation was voluntary and without compensation. Informed written consent was obtained from all participants before enrollment in the study. Details concerning study protocol, inclusion exclusion criteria and surgical procedure are included in the Supplement.

### CPB patient’s blood samples collection and analyses

CPB patient’s blood was drawn pre-anesthesia (P), within 30 minutes of patient’s arrival in the recovery room (RR), and in the early hours of day 1 post-operatively (D1). Additional daily samples were collected from 4 randomly selected patients in the early hours of days 2, 3, 4 (D2, D3, D4). IL-6 was measured using Quantikine ELISA kits, Cat# D6050 (R&D systems, Minneapolis, MN). Insulin was measured using an ELISA assay (sensitivity 2–200 microU/ml; Millipore).

### Antibodies and pharmacologic response modifiers

Antibodies used: Actin (A2066; 1:1000) was from Sigma. Heme oxygenase 2 (HO-2) (PB9213; 1:500) was from Boster Biological Tech Co., Pleasanton CA. HIF-1α (sc-10790; 1:250), AMPKα (sc-25792; 1:1000), MMP9 (sc-10737; 1:1000), TLR4 (sc-10741; 1:500), BVRA (sc-393385; 1:1000), Akt1 (sc-5928; 1:500), p-LKB1 Ser431 (sc-271924; 1:500), LKB1 (sc-32245; 1:500), p-CaMKKβ Thr286 (sc-32289; 1:750), CaMKKβ (sc-100364; 1:1000), p-mTOR Ser2481 (sc-293132; 1:1000), mTOR (sc-517464; 1:1000), p-PKCζ Thr410 (sc-271962; 1:500), PKCζ (sc-393218; 1:500), heme oxygenase 1 (HO-1) (sc-136960; 1:1000), heme oxygenase 2 (HO-2) (sc-17786; 1:1000), tuberin (TSC1/2)(sc-271314; 1:500), haptoglobin β (sc-390962; 1:500), p-NOS3 Ser1177 (sc-81510; 1:500) and NOS3 (sc-376751; 1:500) were all from Santa Cruz Biotechnology. p-Raptor Ser792 (#2083; 1:1000), Raptor (#2280; 1:200), p-p70 S6 kinase Thr389 (#9205; 1:1000), p-AMPKα Thr172 (#2535; 1:1000), p-Akt Ser473 (#4051; 1:1000), p-Akt Thr308 (#9275; 1:1000), tuberin/TSC2 (#4308; 1:1000) and p-tuberin/TSC2 Ser1387 (#5584; 1:1000) were all from Cell Signaling Technology.

Reagents, source and final concentrations (unless otherwise indicated): LPS (lipopolysaccharide from *Eschericnia coli* 0111:B4; Sigma) (blood samples were generally treated with 10 ng/ml and Raw 264.7 cells with 100 ng/ml). Biliverdin (50 μM) (Sigma) was freshly dissolved in 0.2 N NaOH, adjusted to a final pH of 7.4 with HCl and kept in the dark. Metformin (10 μM) was from Sigma. GSK2334470 (3 μM) was from Cayman. Rapamycin (100 nM), torin (50 nM), PP242 (100 nM) were from Tocris. The PKCζ inhibitory peptide (ζ-pseudosubstrate inhibitory peptide) (10 μM) was from Fisher Scientifics.

### *In vitro* studies

Blood drawn into heparin-containing tubes was separated into aliquots and treated with LPS (10 ng/ml) or biliverdin (50 μM), unless otherwise indicated. Leukocytes were then isolated as described^19^. In some experiments, neutrophils and mononucleated cells (which include monocytes and lymphocytes) were isolated, treated or not, and lysed as described^10^. Raw 264.7 cells were obtained from ATCC and cultured for up to 5 passages. Cell lysates were normalized for protein content and analyzed by western blotting. In brief, samples were subjected to SDS-PAGE separation followed by blotting onto polyvinylidene difluoride membrane. Immunoreactive bands were detected using Super Signal Chemiluminescence (Thermo Scientific Pierce) and visualized by autoradiography. All figures shown are an accurate representation of the data and no image was manipulated.

### Immunoprecipitation

Pierce crosslink IP kit (Prod #26147, Thermo Scientific) was used to crosslink BVRA antibody to agarose. Leukocyte pellets were washed once with PBS and then lysed for 5 min on ice with 500 μl of RIPA buffer. Cellular debris was removed by centrifugation and supernatants were normalized for protein concentration. Lysates (500 μg protein) were pre-cleared for 2 h at 4 °C with 40 μl of protein A/G agarose beads and were then incubated overnight at 4 °C with 2 μg of BVRA-agarose with gentle rotation. Samples were then washed three times with washing buffer (0.025 M Tris, 0.15 M NaCl, 0.001 M EDTA, 1% NP-40, 5% glycerol; pH 7.4). Bound proteins were eluted with 5X NaDodSO_4_ sample buffer and analyzed by western blotting as described earlier.

### Statistical analyses

Clinical and laboratory data were analyzed with respect to the detection of TLR4-SI on day 1 post-CPB. *t*-test was used to analyze continuous variables and Mann-Whitney test for comparison of non-parametric data. Categorical variables were compared using Fisher exact test. Normality was determined using D’Agostino and Pearson test. Demographic and clinical variables recognized in unadjusted comparisons as statistically significant at *p-*value of less than 0.1 were selected for inclusion in a logistic regression model. Data are presented as mean and 95% confidence interval (95%CI) of mean if data were normally distributed, or median and interquartile (Q25, Q75%) range if data were not normally distributed. Within group values were compared using ANOVA with repeated measure and Tukey’s multiple comparisons test, or Friedman test and Dunn’s multiple comparisons test if data were not normally distribution. Data and statistical analyses were performed using Prism 7.0 for Mac OSX (Graph Pad software, Inc) and STATISTICA 12.0 for Windows (StatSoft Inc. Oklahoma, USA). *p*-values less than 0.05 were considered statistically significant.

## Results

### Expression of TLR4 signaling intermediates in leukocytes of patients after CPB is inversely correlated with preoperative serum bilirubin levels

**Figure 1:**
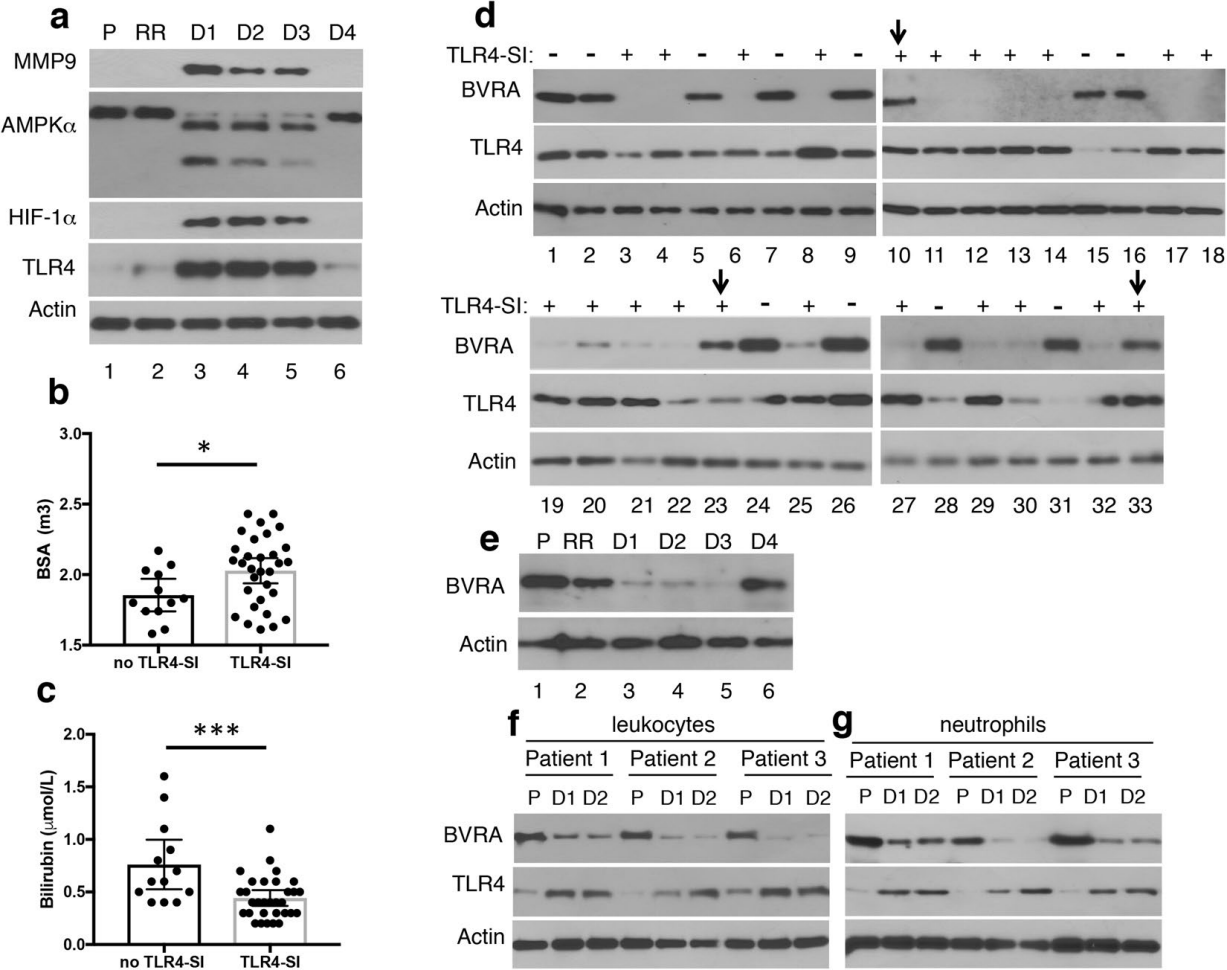
Expression of TLR4 signaling intermediates in leukocytes of patients after CPB is inversely correlated with preoperative serum bilirubin levels and BVRA expression in leukocytes. (**a**) CPB patient’s blood was drawn preoperatively (P), in the recovery room (RR), and on days 1–4 (D1-D4) postoperatively. Leukocytes of 31 of the 44 patients studied exhibited the expression pattern shown in (**a**). The remaining 11 patients did not express TLR4-SI postoperatively. (**b**,**c**) Patient’s data were analysed with respect to the expression of TLR4-SI postoperatively. The data are reported in Table 2. Patients expressing TLR4-SI had (**b**) significantly higher body surface area (BSA; p = 0.028) and (**c**) significantly lower bilirubin (p = 0.003). (**d**) Preoperative CPB patient’s leukocytes were analyzed for BVRA and TLR4 expression. Data were evaluated with respect to the expression of TLR4-SI postoperatively. (−) and (+) identify, respectively, patients who did not express or expressed TLR4-SI postoperatively. Downward arrows identify several patients who expressed TLR4-SI postoperatively despite high preoperative BVRA expression. (**e**) CPB patient’s leukocytes studied in (**a**) were analyzed for BVRA expression. (**f**,**g**) Blood drawn from 3 randomly selected CPB patients preoperatively (P) and on postoperative days 1–2 (D1-D2) was used for parallel (**f**) leukocytes and (**g**) neutrophils isolation and analyses. In (**a**,**d–g**) lysates normalized for protein content were analyzed by western blotting. Actin served as a control for equal protein loading.

We conducted a prospective blinded study of a cohort of patients scheduled for elective CPB. Patient’s demographics are presented in Table 1. Patient’s leukocytes were analyzed for expression of previously described TLR4 signaling intermediates (SI), referred to as ‘TLR4-SI’, which include MMP9, AMPKα, HIF-1α and TLR4^9^. MMP9, HIF-1α and TLR4 expression was low and AMPKα was intact in leukocytes of patients prior to or immediately after CPB (Fig. 1a, lanes 1 and 2). However, leukocytes of 31 of the 44 patients (70%) exhibited increased MMP9, HIF-1α and TLR4 expression and proteolytically cleaved AMPKα by day 1 postoperatively (Fig. 1a). A similar expression pattern was seen for up to day 4 postoperatively (Fig. 1a).

**Table 1:** Cardiopulmonary bypass (CPB) patient’s demographics and blood chemistries.

Patients#	44
Sex (M)	26
Age (y)	69 (66, 73)
BMI (kg/m^2^)	30 (29, 32)
BSA (m^2^)	2 (1.9, 2.1)
HbA1C (%)	5.8 (5.7, 5.9)
Fasting glucose (mg/dL)*	110 (102, 123)
Fasting Insulin (µU/mL)*	3.8 (2.7, 4.9)
ALT (IU/L)*	21 (13, 26)
AST (IU/L)*	21 (16, 29)
Bilirubin (µmol/L)*	0.5 (0.3, 0.6)
Cholesterol (mg/dL)*	164 (148, 192)
Creatinine (mg/dL)	0.9 (0.85, 1)
HDL (mg/dL)*	50 (41, 60)
Triglyceride (mg/dL)*	108 (77, 136)
LDL (mg/dL)*	93 (77, 106)

Data are presented as mean and 95% confidence interval (95% CI) of mean, or as median and interquartile (Q25, Q75%) range, indicated by*. BMI (body mass index), BSA (body surface area), HbA1C (Hemoglobin A1C), AST (aspartate transaminase), ALT (alanine transaminase), HDL (high density lipoprotein), LDL (low density lipoprotein).

Next, patient’s demographics, clinical parameters, and blood chemistries were all analyzed with respect to the expression of TLR4-SI postoperatively (Table 2). Patients expressing TLR4-SI had significantly higher (*p* = 0.028) body surface area (BSA) (Fig. 1b), but not body mass index (BMI). Patient’s gender, BSA and BMI are presented in Supplementary Table 1. In addition, patients expressing TLR4-SI had significantly lower (*p* = 0.003) bilirubin (Fig. 1c) and insulin (*p* = 0.041) than those without TLR4-SI. Those factors showing a bivariate association with the dependent variable, *i.e*., TLR4-SI at *p* < 0.1, were entered into a multivariate logistic regression model. Detection of TLR4-SI was independently associated with decreased levels of total bilirubin [OR 0.02; 95%CI 0.01, 0.55] and increased body surface area [OR, 43.41; 95%CI 1.15, 162.60].

**Table 2:** Cardiopulmonary bypass (CPB) patient’s demographics, and pre-surgery clinical data and blood chemistries analyzed with respect to the expression of leukocytes TLR4 signaling intermediates on postoperative Day 1.

	TLR4-SI positive	TLR4-SI negative	*p* value
Patients#	31	13	
Sex (M)	18	8	
Age (y)	67.5 (63.1, 71.8)	71.9 (66.4, 77.5)	0.2
BMI (kg/m^2^)	30.4 (28.2, 32.6)	28.3 (25.4, 31.2)	0.3
BSA (m^2^)	2.0 (1.9, 2.1)	1.8 (1.7, 1.9)	<0.03
HbA1C (%)	5.8 (5.6, 5.9)	5.7 (5.5, 6.1)	0.4
Fasting glucose (mg/dL)	118 (111, 126)	111 (99, 123)	0.3
Fasting Insulin (µU/mL)*	4.0 (2.7, 4.3)	5.7 (4.0, 7.3)	0.1
ALT (IU/L)*	20 (13, 29)	22 (15, 34)	0.4
AST (IU/L)*	20 (16, 29)	23 (1, 37)	0.1
Bilirubin (µmol/L)*	0.4 (0.3, 0.5)	0.7 (0.5, 1.0)	<0.01
Cholesterol (mg/dL)*	164 (146, 204)	156 (136, 179)	0.4
Creatinine (mg/dL)	0.9 (0.8, 1.0)	0.9 (0.7, 1.3)	0.1
HDL (mg/dL)*	50 (41, 63)	45 (34, 54.)	0.2
Triglyceride (mg/dL)*	107 (78, 143)	112 (80, 135)	1.0
LDL (mg/dL)	105 (90, 119)	83 (67, 100)	0.1
IL-6 (pg/ml)*	3 (1, 9)	2 (1, 5)	0.7
CRP (ng/ml)*	2.9 (0.9, 3.7)	2.2 (0.5, 8.4)	0.8

Data are presented as mean and 95% confidence interval (95%CI) of mean, or as median and percentiles ((Q25, Q75%; marked by (*)). Data were compared using *t*-test or Mann-Whitney U test. A *p*-value < 0.05 was considered statistically significant.

Biliverdin reductase A (BVRA), the enzyme that reduces biliverdin to bilirubin, is ubiquitously expressed. BVRA expression in CPB patients leukocytes obtained on the morning of the surgery varied among patients (Fig. 1d). Strikingly, BVRA expression in leukocytes of patients who did not express TLR4-SI postoperatively was in general higher than in leukocytes of patients who expressed TLR4-SI postoperatively (see Supplementary Table 1 for patient’s demographics). In addition, whereas TLR4-SI expression postoperatively increased, BVRA expression transiently decreased (Fig. 1e). Preoperative BVRA expression levels and/or presence of TLR4-SI postoperatively did not correlate with patient’s intensive care unit length of stay and/or time to discharge.

Neutrophils constitute >60% and lymphocytes ~30% of all leukocytes in humans blood. Naïve T and B cells are less responsive to LPS than neutrophils^20,21^. Analyses of leukocytes and neutrophils from three randomly selected CPB patients showed that the transient decline in BVRA expression seen in leukocytes was reproduced in neutrophils (Fig. 1f,g). Together, the data established that postoperative TLR4-SI expression in CPB patient’s leukocytes/neutrophils was inversely associated with both preoperative serum bilirubin and BVRA expression, suggesting that biliverdin/BVRA may counter-regulate TLR4 signaling.

### Biliverdin activates mTORC2 and AMPK signaling in leukocytes

**Figure 2:**
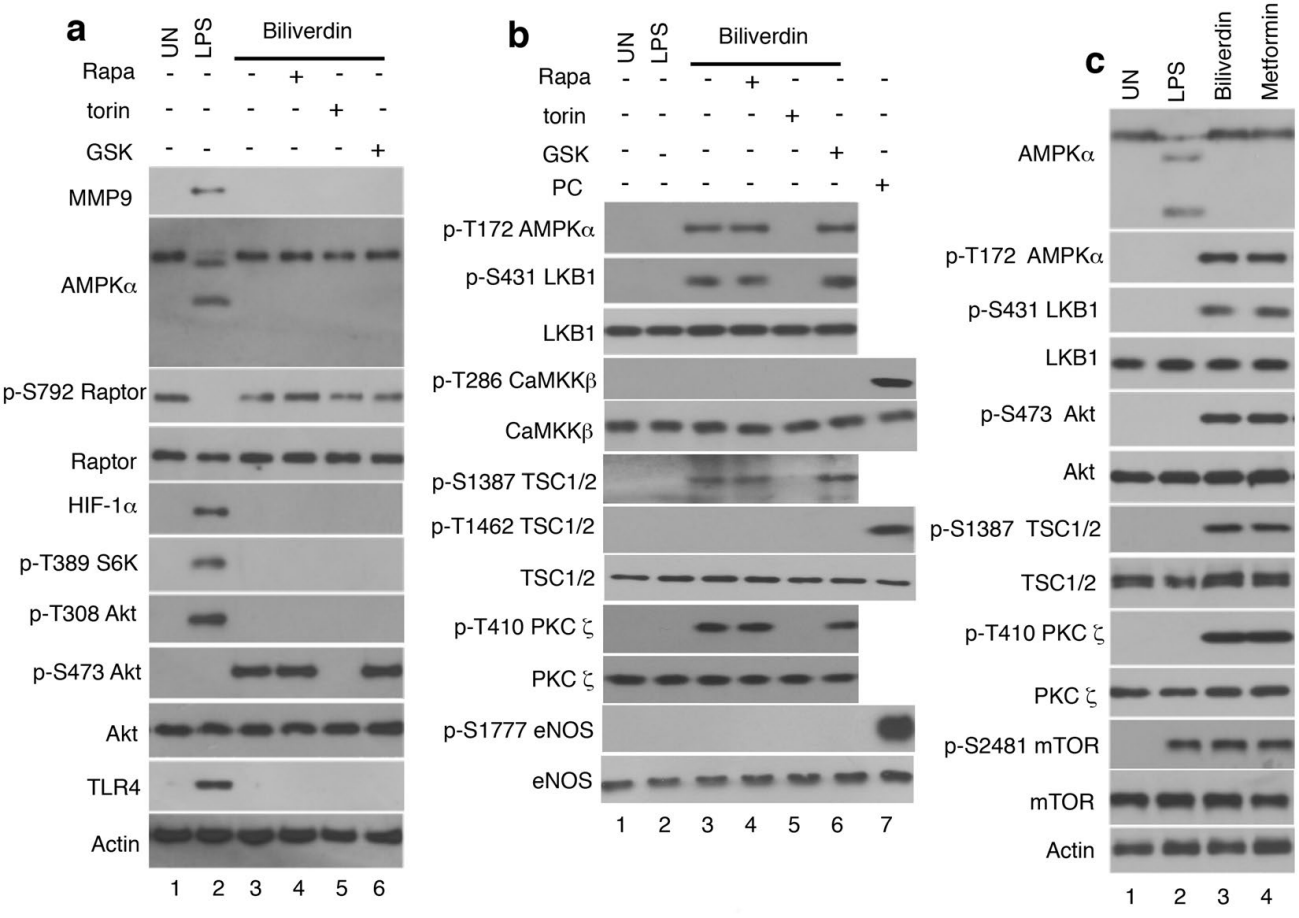
Biliverdin activates mTORC2 and AMPK signaling in leukocytes. Healthy donor’s blood was untreated or treated *in vitro*. Then, leukocytes were isolated, lysed, normalized for protein content and analyzed by western blotting. (**a**,**b**) Blood was untreated (UN; lane 1), or treated for 1 hr with LPS alone (10 ng/ml; lane 2), biliverdin alone (50 μM; lane 3), or co-treated with biliverdin plus rapamycin (Rapa, 100 nM; lane 4), plus torin (50 nM; lane 5), or plus GSK2334470 (GSK, 3 μM; lane 6). In (**b**). lane 7, a lysate of biliverdin treated (50 μM; 1 hr) Raw 264.7 cells served as a positive control (PC). (**c**) Blood was untreated (UN; lane 1), or treated for 1 hr with LPS (10 ng/ml; lane 2), biliverdin (50 μM; lane 3), or metformin (10 μM; lane 4).

To clarify the role of biliverdin relative to TLR4 signaling in leukocytes, we first characterized the biliverdin/BVRA signaling pathway in these cells. Biliverdin and LPS activated dissimilar SI (Fig. 2). LPS did^10^, but biliverdin did not induce S6K1 phosphorylation at Ser389 (Fig. 2a), the target of functional mTORC1^16,17^. In addition, as previously reported^10^, LPS triggered Akt phosphorylation at Thr308, but not Ser473 (Fig. 2a), the mTORC2-dependent phosphorylation site^22^. In contrast, biliverdin induced Akt phosphorylation at Ser473 (Fig. 2a). Together, the data suggested that whereas TLR4 activates mTORC1, biliverdin/BVRA activates mTORC2.

AMPKα cleavage is a hallmark of LPS/TLR4 signaling in human leukocytes^8,9^. In contrast, biliverdin induced AMPKα phosphorylation at Thr172 (Fig. 2b). Two kinases, LKB1^23^ and Ca^+2^/calmodulin-dependent kinase kinase β (CaMKΚβ)^24,25^ phosphorylate AMPKα at Thr172. Activated LKB1 is phosphorylated at Ser431^26^ whereas CaMKΚβ autophosphorylates at Thr286^27^. Though both LKB1 and CaMKΚβ where detected in leukocytes, biliverdin induced only LKB1 Ser431 phosphorylation (Fig. 2b). However, as reported^28^, biliverdin induced CaMKΚβ activation in Raw264.7 cells (Supplementary Fig. 1). CaMKΚβ phosphorylates endothelial nitric oxide synthase (eNOS) at Ser1177^28^. eNOS Ser1177 phosphorylation was undetectable in biliverdin-treated leukocytes (Fig. 2b), but as reported^29^, was evident in biliverdin-treated Raw 264.7 cells used here as controls (Fig. 2b lane 7). Others noted differences related to NFκB signaling in Raw264.7 versus HEK293A cells^30^. We also compared leukocyte’s, neutrophil’s and mononucleated cell’s (monocytes and lymphocytes) responses to biliverdin. Reproducing the pattern seen in leukocytes, Akt Ser473, AMPKα Thr172, and LKB1 Ser431 phosphorylation were all detected in neutrophils but not mononucleated cells (Supplementary Fig. 2). These observations and prior data^30^ established that biliverdin/BVRA engages signaling elements that are, at least in part, cell-type specific.

Like biliverdin, metformin, the most widely used drug for treating type 2 diabetics, activates LKB1 and AMPK^31^. Therefore, we assumed that there could be additional targets that both biliverdin and metformin engage. As shown in cardiomyocytes^32^, metformin induced Akt phosphorylation at Ser473 in leukocytes (Fig. 2c). Both metformin^33^ and biliverdin induced tuberous sclerosis complex proteins (TSC1/2 complex) phosphorylation at Ser1387, but not at Thr1462 (Fig. 2b,c). The distinction between Ser1387 and Thr1462 is important; Ser1387 is phosphorylated by AMPK and involved in mTORC1 inhibition^34^ whereas TSC1/2 phosphorylation at Thr1462 is Akt dependent and contributes to mTORC1 activation^13,35,36^. In metformin treated human and bovine umbilical vein endothelial cells, PKCζ contributed to LKB1 Ser428 (Ser431 in humans) and AMPKα Thr172 phosphorylation^37^. Other showed that mTORC2-dependent phosphorylation at Thr410 activates PKCζ^38^. Here, both biliverdin and metformin induced PKCζ phosphorylation at Thr410 (Fig. 2b,c). LPS, metformin, and biliverdin all induced mTOR Ser2481 phosphorylation, which is associated with both mTORC1 and mTORC2^39^ (Fig. 2c). The data established that multiple SI implicated in mTORC2 and AMPK activation, and on the other hand, mTORC1 inhibition are associated with metformin and biliverdin signaling in leukocytes.

Leukocytes are not amenable to genetic manipulations. Therefore, to further examine the role of mTORC2 relative to biliverdin signaling in leukocytes, blood samples were co-treated with biliverdin and one of three well-characterized pharmacologic response modifiers: rapamycin, an acute mTORC1 inhibitor, torin, an mTORC1 and mTORC2 inhibitor^40^, and GSK2334470 (GSK), an inhibitor of 3-phosphoinositde-dependent protein kinase (PDK1)^41^. In LPS treated leukocytes (Supplementary Fig. 3), rapamycin and torin inhibited HIF-1α expression and S6K1 phosphorylation at Ser389; GSK also inhibited Akt phosphorylation at Thr308. In marked contrast, while rapamycin and GSK had not effect, torin inhibited all biliverdin-induced responses (Fig. 2a,b). Taken together, the data established that mTORC2 is central to biliverdin signaling in leukocytes.

### Biliverdin inhibits TLR4 signaling in leukocytes via mTORC2

**Figure 3:**
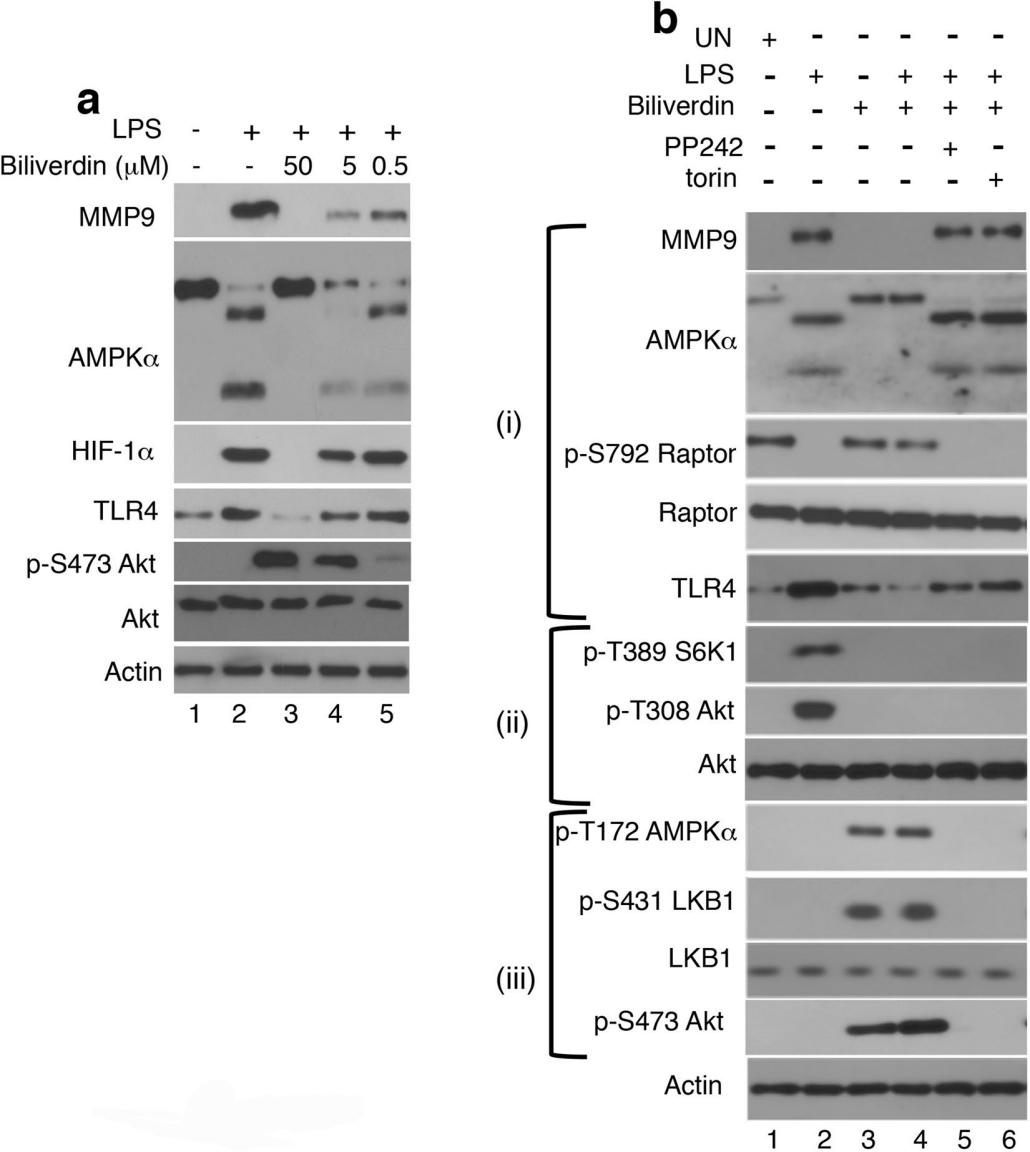
Biliverdin inhibits TLR4 signalling in leukocytes via mTORC2. Healthy donor’s blood was untreated or treated *in vitro*. Leukocytes were isolated, lysed, normalized for protein content and analyzed by western blotting. (**a**) Blood was untreated (lane 1), or treated for 1 hour with LPS (10 ng/ml) (lane 2) or LPS plus biliverdin (lanes 3–5) at the indicated concentration. (**b**) Blood was untreated (UN; lane 1), or treated for 1 hr with LPS alone (10 ng/ml; lane 2), biliverdin alone (50 μM; lane 3), or co-treated for 1 hr with LPS plus biliverdin (lane 4), LPS plus biliverdin and PP242 (100 nM; lane 5), or LPS plus biliverdin and torin (50 nM; lane 6). Based on our recently proposed model^10^, bracket (i) denotes LPS/TLR4 signaling intermediates that are upstream to and required for mTORC1 activation, whereas bracket (ii) denotes LPS/TLR4 signaling intermediates that are downstream to and dependent on mTORC1. Bracket (iii) denotes biliverdin-dependent signaling intermediates.

Next, we examined the role of biliverdin relative to TLR4 signaling in leukocytes. As demonstrated in Raw264.7 cells^28^, biliverdin inhibited TLR4 signaling in leukocytes in a dose-dependent manner (Fig. 3a). In LPS-treated leukocytes, the increase in MMP9 and TLR4 expression, AMPKα cleavage and Raptor dephosphorylation at Ser792 are all upstream to mTORC1^10^. Therefore, by blocking BVRA-mTORC2 signaling, PP242 and torin, chemically distinct ATP-competitive inhibitors of mTORC1 and mTORC2^40,42^, reversed the effect of biliverdin and restored the expression pattern of SI associated with TLR4 signaling ((Fig. 3b (i)). In contrast, mTORC1 phosphorylates S6K1 at Ser389 and triggers, though indirectly, Akt phosphorylation at Thr308^10^. Thus, since torin and PP242 blocked both BVRA-mTORC2 and TLR4-mTORC1 signaling, S6K1 and Akt phosphorylation remained suppressed in leukocytes co-treated with biliverdin and LPS ((Fig. 3b (ii)). Torin and PP242 inhibited AMPKα Thr172, LKB1 Ser431, and Akt Ser473 phosphorylation ((Fig. 2c, (iii)). The data suggested that mTORC2 plays a central role relative to biliverdin-inhibition of TLR4.

### mTORC2 is a key regulator of biliverdin/BVRA intermolecular interactions

**Figure 4:**
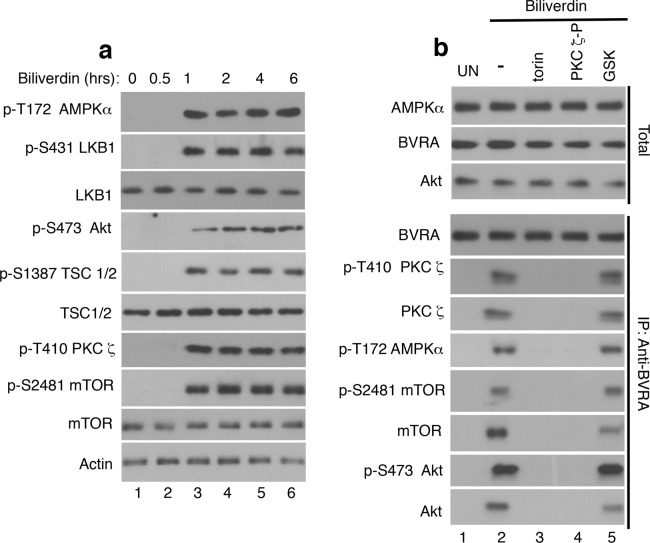
mTORC2 is a regulator of BVRA intermolecular interactions. (**a**) Healthy donor’s blood was untreated (lane 1) or treated with biliverdin (50 μM; lane 2–6) for the indicated times. Leukocytes were isolated, lysed, normalized for protein content and analyzed by western blotting. (**b**) Healthy donor’s blood was untreated (UN; lane 1) or treated for 1 with biliverdin alone (50 μM; lane 2), biliverdin plus torin (50 nM; lane 3), plus PKCζ inhibitory peptide (PKCζ-P) (10 μM; lane 4), or plus GSK2334470 (GSK, 3 μM; lane 5). Leukocytes were isolated and lysed. Lysate containing equal total protein amounts (Total) were analyzed by western blotting or were subjected to BVRA-immunoprecipitation (IP). BVRA co-immunoprecipitated proteins were detected by western blotting.

In leukocytes and neutrophils treated with LPS, SI appeared within 10–90 min^9^. In contrast, time-course studies conducted in biliverdin-treated leukocytes (Fig. 4a) revealed co-appearance of all phosphorylated proteins studied by 60 minutes. Numerous BVRA interacting proteins were identified^43–45^. This suggested the hypothesis that it may take approximately 60 min for BVRA to establish stable intermolecular interactions. Then, we sought to determine which of the SI activated in biliverdin treated leukocytes interact with endogenous BVRA. To this end, untreated and biliverdin-treated leukocytes were lysed and subjected to BVRA immunoprecipitation followed by western blotting to identify interacting proteins (Fig. 4b). Phosphorylated LKB1, AMPKα, PKCζ, Akt and mTOR were all detected in biliverdin-treated leukocyte’s co-immunoprecipitates (Fig. 4b). Torin^37^, but not GSK2334470, the PDK1 inhibitor, prevented all BVRA’s intermolecular interactions (Fig. 4b). A well-characterized PKCζ inhibitory peptide previously used to determine the role of PKCζ in neutrophils^46^ also prevented complexes formation. The data established that BVRA interacts either directly or indirectly with multiple SI in biliverdin treated leukocytes. mTORC2 and PKCζ may facilitate formation of and/or stabilize BVRA complexes once formed.

### TLR4 is a negative regulator of biliverdin/BVRA signaling

**Figure 5:**
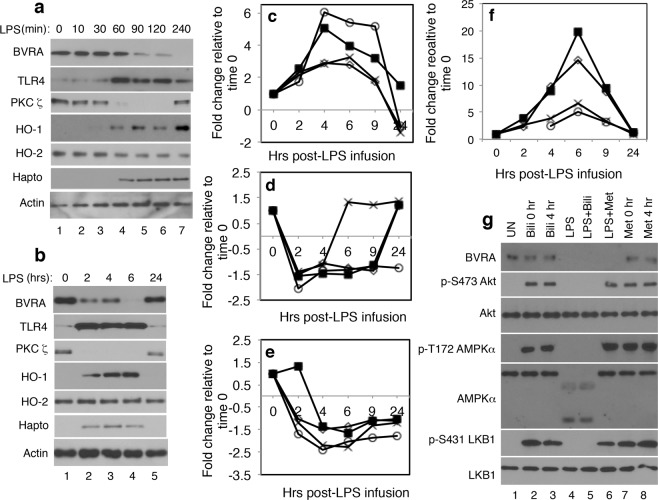
TLR4 is a negative regulator of biliverdin/BVRA signaling. (**a**) Healthy donor’s blood was untreated (lane 1) or treated with LPS (10 ng/ml) for the indicated times (lanes 2–7). Leukocytes were isolated and analyzed. Abbreviations are: Heme oxygenase 1, HO-1; Heme oxygenase 2, HO-2; Haptoglobin, Hapto. (**b**) In an earlier study^8^, subjects were administered LPS (1 ng/kg) *in vivo* and blood was drawn at the indicated times post LPS infusion. Leukocyte lysates available from that study were normalized for protein content and analyzed by western blotting. (**c**–**f**) In another prior study^7^, leukocytes from four subjects administered LPS *in vivo* were analyzed for changes in gene expression over a period of 24 hours post LPS infusion. Data from that study^7^, available through GEO dataset GSE3284, revealed a temporal (**c**) increase in TLR4 mRNA, (**d**) decline in BVRA mRNA, (**e**) decrease in PKCζ mRNA (**f**) increase in haptoglobin mRNA expression. In (**c**–**f**) each symbol represents a subject. (**g**) At time 0 (0 hr) healthy donor’s blood was untreated (UN; lane 1), treated for 1 hour with biliverdin (Bili 0 hr; 50 μM; lane 2), treated for 4 hours with LPS (10 ng/ml; lanes 4–6) to trigger a decline in BVRA expression, or for 1 hour with metformin (Met; 10 μM; lane 7). Four hours later (time 4 hr) blood samples were treated for 1 hour with biliverdin (Bili 4 hr; 50 μM; lane 3) or metformin (Met 4 hr; 10 μM; lane 8). Samples pretreated with LPS for 4 hours (lanes 4–6), were then treated for 1 hr with biliverdin (Bili; 50 μM; lane 5) or metformin (Met; 10 μM; lane 6). Leukocytes were isolated and analyzed by western blotting.

Having found that biliverdin/BVRA inhibits TLR4 signaling, the inverse correlation between TLR4-SI and BVRA expression in CPB patient’s leukocytes described earlier (Fig. 1) required explanation. We hypothesized that TLR4 counter-regulates biliverdin/BVRA signaling. In line with this, we found that LPS triggered a decline in BVRA expression in leukocytes *in vitro* (Fig. 5a). Inverse TLR4 and BVRA expression trends were also detected in leukocytes of subjects challenged with a bolus dose (1 ng/kg) of LPS *in vivo*^8^ (Fig. 5b). As reported^47^, total BVRA expression remained unchanged in LPS-treated Raw 264.7 cells (Supplementary Fig. 4a). LPS also did not induce an increase in MMP9 expression, AMPKα cleavage or Raptor dephosphorylation at Ser792 in Raw264.7 cells (Supplementary Fig. 4b), establishing that LPS and biliverdin signaling in human leukocytes differ from those previously documented in Raw264.7 cells.

In an earlier study^7^, leukocytes of subjects challenged with LPS *in vivo* were subjected to genome-wide transcriptome analyses. Data from that study, available through GEO Dataset GSE3284, showed that LPS-induced an increase in TLR4 mRNA (Fig. 5c), and decreases in BVRA (Fig. 5d) and PKCζ (Fig. 5e) mRNA expression. Consistent with the latter, LPS triggered a decrease in PKCζ protein expression in leukocytes *in vitro* (Fig. 5a) and *in vivo* (Fig. 5b). Li and Gao reported^38^ that following phosphorylation by mTORC2, PKCζ was more resistant to proteosomal degradation. Therefore, it is possible that in addition to the decline in transcriptional expression, mTORC2 inhibition in LPS treated leukocytes contributed to unphosphorylated PKCζ degradation.

Heme oxygenase(s) (HO) convert heme to biliverdin, the substrate of BVRA. Two HO isoforms were described in leukocytes: HO-1 and HO-2. The level of HO-1 is resting leukocytes was below detection (Fig. 5), suggesting that biliverdin production at steady state is primarily HO-2 dependent. Consistent with this, others demonstrated that bilirubin production in mice neuronal cells required HO-2 but not HO-1^48^. As in macrophages^49,50^, LPS triggered an increase in HO-1 expression, but not HO-2, in leukocytes *in vitro* and *in vivo* (Fig. 5a,b). Despite evidence that HO-1 may regulate interferon beta production in LPS treated bone-marrow derived macrophages^51^, the role of HO-1 in leukocytes remains unclear. Furthermore, contrary to the prevailing notion that HO-1 has an anti-inflammatory function, knockout of HO-1 in mice liver and myeloid cells contributed to a decrease in mice susceptibility to diet-induced insulin resistance and inflammation^52^. As proposed^52^, it is possible that HO-1 drives rather than inhibits inflammation in the context of metabolic diseases.

**Figure 6:**
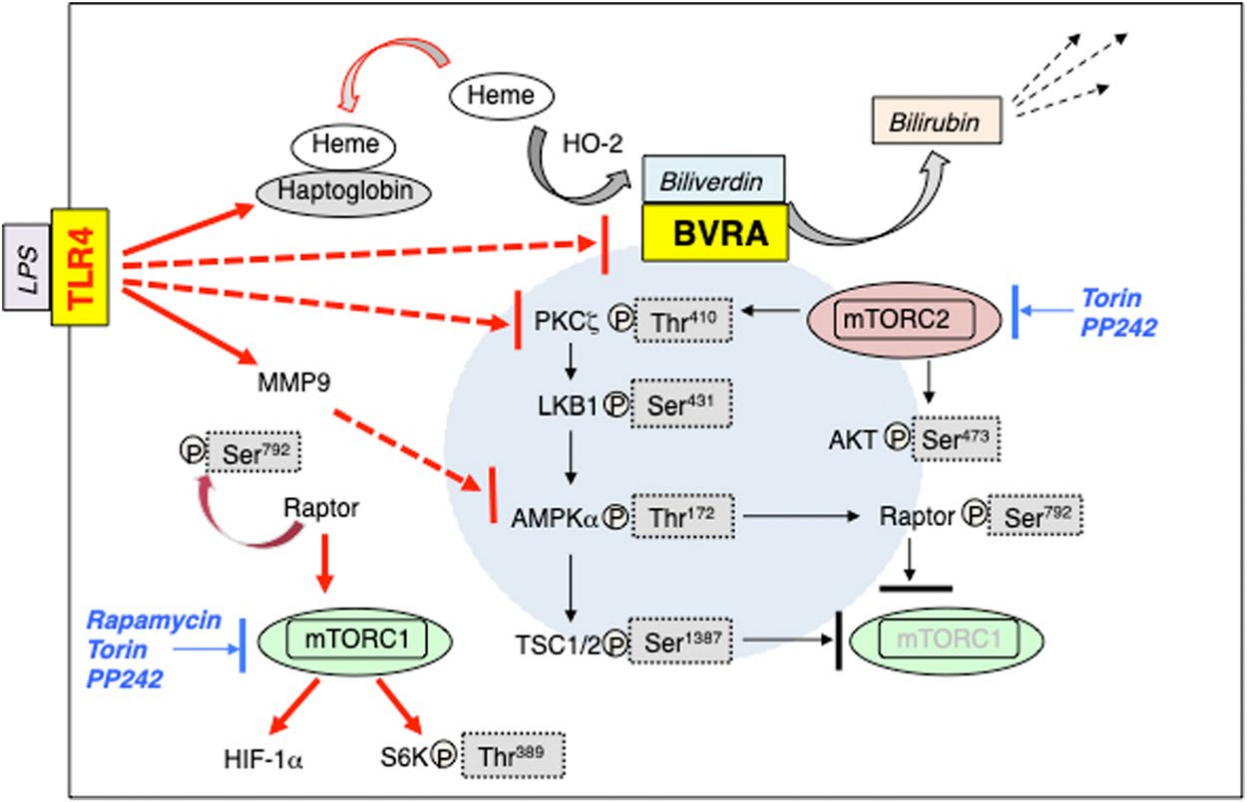
Our working model for biliverdin/BVRA and TLR4 signaling in leukocytes. Biliverdin induced responses are shown in black. Biliverdin acts on BVRA. Our data suggest that biliverdin facilitates formation of BVRA signaling complex(s) that include mTORC2. mTORC2 induces phosphorylation of two targets: PKCζ at Thr410 and Akt at Ser473. PKCζ is involved in LKB1 Ser431 and AMPKα Thr172 phosphorylation. AMPK phosphorylates TSC1/2 at Ser1387 and Raptor at Ser792. Therefore, BVRA activation may contribute to mTORC1 inhibition via two SI: phosphorylated Raptor, which inhibits mTORC1 complex formation, and TSC1/2 mediated inhibition of mTORC1 activity. Because mTORC1 is central to TLR4 signaling in leukocytes^10^, BVRA inhibition of mTORC1 suppresses TLR4. Pharmacological inhibitors are shown in blue. Torin and PP242, but not rapamycin, inhibited BVRA-mediated protein-protein interactions. TLR4 induced response are shown in red. Once activated, TLR4 down-regulates BVRA and PKCζ expression (dashed lines). TLR4 upregulates expression of MMP9^9^, which then triggers AMPKα cleavage (dashed line), leading to Raptor Ser792 dephosphorylation^9^. This enables mTORC1 activation, which then triggers an increase in HIF-1α expression and S6K1 phosphorylation at Thr389. TLR4 also triggers an increase in haptoglobin expression, thus limiting heme availability. Using these parallel mechanisms TLR4 ensures biliverdin/BVRA and mTORC2 signaling inhibition, and on the other hand, mTORC1 activation.

Haptoglobin is a high-affinity heme binding protein that regulates heme availability. A recent study found that monocytes of subjects with Gilbert’s syndrome, a syndrome associated with higher than normal bilirubin levels, had significantly lower intracellular haptoglobin levels as compared to matched controls^53^. Neutrophils synthesize and store haptoglobin in their granules and release it following activation^54^. Leukocytes treated with LPS *in vitro* (Fig. 5a) and *in vivo* (Fig. 5b) exhibited elevated intracellular haptoglobin protein expression. Data from GEO Dataset GSE3284 showed that LPS also triggered an increase in haptoglobin mRNA in leukocytes *in vivo* (Fig. 5f). Therefore, TLR4 may deregulate biliverdin/BVRA signaling in leukocytes by suppressing expression of pathway SI and heme availability. Our work model, summarizing both BVRA and TLR4 signaling in leukocytes, is presented in Fig. 6.

Can a decline in BVRA expression alter leukocyte’s sensitivity to biliverdin? To address this question, leukocytes were first treated for 4 hours with LPS to induce a decline in BVRA expression (Fig. 5g lanes 4–6). Then, leukocytes were treated for 1 hour with biliverdin (Fig. 5g lane 5) or metformin (Fig. 5g lane 6). Absence of Akt Ser473, AMPKα Thr172, and LKB1 Ser431 phosphorylation all indicated that LPS blunted leukocyte’s responses to biliverdin. In contrast, leukocytes pretreated with LPS remained responsive to metformin. These findings suggested that leukocytes with reduced BVRA expression are less responsive to biliverdin.

### Expression of BVRA signaling intermediates in T2D patient’s leukocytes

**Figure 7:**
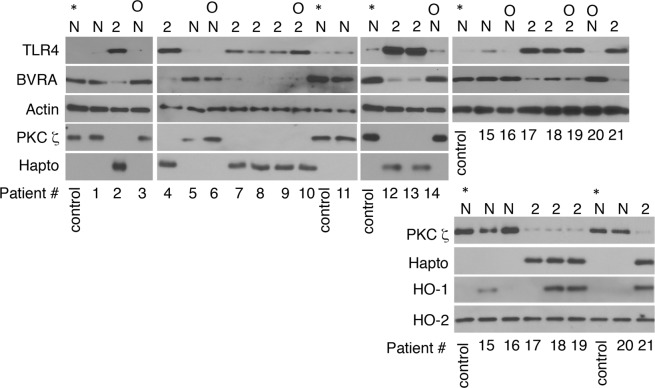
Expression of BVRA signaling intermediates in T2D patient’s leukocytes. Leukocytes obtained from a previously described cohort of patients without diabetes (N) or with T2D (2)^10^ were isolated and analyzed by western blotting. (O), obese patients (BMI > 30). *A healthy control sample used multiple times.

Then, we sought to explore whether our findings had clinical correlates. We reported that leukocytes of patients with T2D expressed TLR4-SI^9^. Here, using TLR4 as a surrogate for TLR4-SI expression, leukocytes of a previously described cohort of patients without or with T2D^10^ were analyzed for BVRA, PKCζ and haptoglobin expression (Fig. 7). Reproducing the pattern seen in LPS-treated leukocytes (Fig. 5), leukocytes of patients with T2D exhibited reduced BVRA and PKCζ and increased haptoglobin expression. Consistent with increased HO-1 expression in liver and visceral fat of obese, insulin resistant subjects^52^, leukocytes of patients with T2D exhibited elevated HO-1 expression. Collectively, the data suggested that biliverdin/BVRA signaling is deregulated in T2D patient’s leukocytes.

## Discussion

Leukocytes form the first line of defense against invading microorganisms. In humans, minute LPS concentrations and multiple endogenous ligands that are produced by damaged tissues activate leukocyte’s TLR4. Since activated leukocytes may cause sever damage to hosts, we hypothesized existence of physiological mechanisms that prevent unwarranted TLR4 activation, and furthermore, that failure of these mechanisms may contribute to chronic, low-grade inflammatory diseases. We recently reported that insulin is a TLR4-signaling regulator/inhibitor in leukocytes^10^. In this study, analyses of CPB patient leukocytes and leukocytes exposed to LPS *in vitro* and *in vivo* uncovered an additional TLR4 regulatory mechanism that is controlled by biliverdin and BVRA. We found that biliverdin/BVRA inhibits TLR4 signaling using multiple SI, including PKCζ, LKB1, AMPK, TSC1/2 and Akt. We conclude that mTORC2, but not mTORC1, is central to biliverdin/BVRA signaling in human leukocytes. Two observations support this conclusion: (i) biliverdin induced Akt phosphorylation at Thr473, the mTORC2 phosphorylation site^22,55^, but failed to induce S6K1 phosphorylation at Ser389, the mTORC1 phosphorylation site^16^, and (ii) torin and PP242, which act on mTORC1 and mTORC2, suppressed responses to biliverdin, whereas rapamycin, which under acute conditions inhibits only mTORC1, did not. These findings are significant since they demonstrate that bilirubin/BVRA inhibits TLR4/mTORC1 signaling in leukocytes via mTORC2. A similar mechanism may prevent TLR4 activation at steady state.

It is interesting to compare the mechanisms by which insulin and biliverdin/BVRA inhibit TLR4 signaling in leukocytes. Contrasting biliverdin/BVRA signaling dependence on mTORC2, insulin signaling in leukocytes, and in general, involves both mTORC1 and mTORC2 activation^10^. Consequently, whereas PKCζ-LKB1-AMPK-TSC1/2 inhibited mTORC1 in the context of biliverdin/BVRA signaling in leukocytes, insulin suppressed TLR4 signaling via mTORC2-Akt Ser473 and Foxo1/3 without LKB1-AMPK-TSC1/2 involvement. These data demonstrate that mTORC2 can engage distinct SI subsets to inhibit TLR4. Whereas the insulin and biliverdin/BVRA signaling pathways that converge on mTORC1 differ, we noted striking mechanistic similarities between biliverdin/BVRA and metformin signaling in leukocytes. As one of the oldest and most commonly used drugs for treating type 2 diabetics, metformin’s mechanism of action has been studied extensively. In general, metformin activates LKB1-AMPK-TSC1/2 signaling^33^. Our data now demonstrate that both metformin and biliverdin use mTORC2 to inhibit mTORC1. How biliverdin/BVRA activates mTORC2 remains unanswered. Maines and colleagues suggested that BVRA acts as a scaffold that supports oligomers formation^45,56^. Our data support this possibility and suggest that in leukocytes, BVRA facilitates formation of signaling complexes that include mTORC2 and it’s signaling targets.

Earlier studies have focused on the possibility that bilirubin and biliverdin act primarily as antioxidants and may reduce oxidative stress damage by suppressing intracellular oxides levels^57,58^. Consistent with this possibility, a recent study demonstrated an increase in oxidative stress indicators in BVRA KO mice^59^. Others reported that bilirubin is a ligand of PPARα, a regulator of lipids metabolism in mice livers^60^. On the other hand, BVRA was implicated in insulin, IGF-1 and TNF-α signaling^28,43–45,47^. In IGF-1 treated HEK293 cells, recombinant BVRA co-immunoprecipitated with Akt1, Akt2 and PDK1 and facilitated Akt phosphorylation at Thr308, the PDK1 phosphorylation site^44,45^. BVRA also interacted with PKCδ in this model cell system^45^. However, in TNFα treated HEK293 cells, BVRA regulated PKCζ^45^. Of these, only PKCζ activation was reproduced in biliverdin-treated leukocytes. Consistent with our findings, Wegiel and colleagues reported that biliverdin triggered TLR4 signaling inhibition in Raw 264.7 cells^28^. However, the TLR4 inhibitory mechanism in Raw 264.7 cells involved endothelial nitric oxide synthase (eNOS) activation by CaMKΚβ and increased nitric oxide generation. Although we observed similar biliverdin-induced responses in Raw264.7 cells, *i.e*., eNOS and CaMKΚβ activation, biliverdin did not induce CaMKΚβ/eNOS signaling in leukocytes, neutrophils or mononucleated cells. The data establish existence of cell-type specific biliverdin/BVRA signaling mechanism(s), and identify a novel biliverdin/BVRA signaling pathway in leukocytes.

Prior clinical and epidemiologic studies found that low serum bilirubin levels were associated with increased risk of pre-diabetes, T2D, metabolic syndrome, as well as stroke^61–65^. Low serum bilirubin were also shown to correlate with higher C reactive protein levels in patients with T2D and impaired glucose tolerance^66^. It is also known that patients with Gilbert’s syndrome, a syndrome associated with higher than normal bilirubin, have in general a lower incidence of coronary heart disease and arteriosclerosis^67^. Together, these clinical studies suggested that whereas high bilirubin levels are health beneficial, low bilirubin levels could be detrimental to health. We hypothesize that higher bilirubin levels are associated with decreased risk for human diseases, and *vice-a-versa*, since these levels reflect, at least in part, how well the biliverdin/ BVRA signaling network operates. When fully functional, biliverdin//BVRA may contribute to higher serum bilirubin levels and at the same time suppress TLR4 signaling in leukocytes.

Notably, our data are first to show that activated TLR4 can counter-regulate BVRA signaling. One of the key biliverdin/BVRA signaling components targeted by TLR4 is BVRA itself. TLR4 may also disrupt biliverdin/BVRA signaling by reducing AMPKα/AMPK, PKCζ, and heme availability. Significantly, leukocytes of patients with T2D expressed low BVRA and PKCζ levels and elevated haptoglobin, reproducing the expression pattern seen in LPS treated leukocytes. Compared to untreated leukocytes, LPS-treated leukocytes with reduced BVRA expression were less responsive to biliverdin. Higher BVRA expression may enhance leukocyte’s resistance to stressors that act on TLR4, or in other words, that biliverdin/BVRA impact leukocyte’s activation threshold. Our data suggest that activated TLR4 deregulates this safety mechanism.

In summary, we discovered a novel TLR4 and biliverdin/BVRA counter regulatory mechanism that controls TLR4 activation in human leukocytes. Furthermore, our data suggest that the balance between TLR4 and biliverdin/BVRA signaling in leukocytes of patients with T2D is deregulated and chronically shifted in favor of TLR4. A fully functional biliverdin/ BVRA signaling network may, in general, suppress unwarranted TLR4 signaling, whereas suboptimal network activity may fail to prevent TLR4 activation thus contributing to non-resolving chronic low-grade inflammation.

## Supplementary information

Supplement
