# Supplementary material for: TLR4 counteracts BVRA signaling in human leukocytes via differential regulation of AMPK, mTORC1 and mTORC2
Source: Sci Rep. 2019 May 7;9:7020. doi: 10.1038/s41598-019-43347-8 (PMC6504875; doi:10.1038/s41598-019-43347-8)
Supplement: Supplementary file 1 — Supplement [file 41598_2019_43347_MOESM1_ESM.pdf]

TLR4 counteracts BVRA signaling in human leukocytes via differential regulation of AMPK, mTORC1 and mTORC2

Zhiyong Zhang[1], Louis F. Amorosa[2], Anna Petrova[3], Susette Coyle[1], Marie Macor[1], Mohan Nair[1], Leonard Y. Lee[1] and Beatrice Haimovich[1*],

From the [1]Department of Surgery, [2]Department of Medicine, and [3]Department of Pediatrics, Rutgers Robert Wood Johnson Medical School (RWJMS), New Brunswick, NJ USA 08903

	Sex	BSA (m^2)	BMI (kg/m^2)
1	F	1.89	32.8
2	M	1.83	23.4
3	M	1.98	23.0
4	F	1.89	29.7
5	F	1.74	24.2
6	M	2.09	38.3
7	M	1.77	26.0
8	M	2.34	30.8
9	F	2.03	34.4
10	M	1.83	23.4
11	M	1.70	22.0
12	F	2.18	41.8
13	F	1.65	23.8
14	F	2.34	42.2
15	F	2.00	38.7
16	M	1.58	25.5
17	M	2.43	34.6
18	M	2.19	26.0
19	F	1.63	22.2
20	F	1.87	29.0
21	F	2.25	42.2
22	F	2.04	33.4
23	F	1.93	32.8
24	M	2.43	37.6
25	M	2.07	31.4
26	F	1.68	28.8
27	M	1.80	26.0
28	F	1.61	22.7
29	F	1.74	27.8
30	M	2.02	29.7
31	M	2.31	37.2
32	F	1.61	22.7
33	F	2.12	41.5
34	M	2.29	30.8

Supplementary Table 1. Shown are gender, body surface area (BSA), and body mass index (BMI) for the patients studied in Fig. 1d. Patient's ID numbers correspond to those shown in Fig. 1d.

Supplementary Figure Legends

Supp. Figure 1. *Biliverdin signaling in Raw 264.7 cells.* Shown is a western blot of Raw 264.7 cells untreated (lane 1) or treated with biliverdin (50 μM) for the indicated times (lanes 2-6). A positive control (PC) is shown in lane 7.

Supp. Figure 2. *Biliverdin signaling in leukocytes, neutrophils and mononucleated cells.* Leukocytes (Leu), neutrophils (Neu) and mononucleated (Mono) cells isolated as described in the methods section were treated with biliverdin (50 μM) for the indicated times and then analyzed by western blotting. A positive control (PC) is shown in lane 10.

Supp. Figure 3. *LPS and biliverdin treated leukocyte's responses to rapamycin, torin and GSK*
Healthy donor's blood was untreated (lane 1), or treated for 1 hour with LPS (10 ng/ml) (lane 2), or LPS plus rapamycin (Rapa, 100 nM; lane 3), torin (50 nM; lane 4) or GSK2334470 (GSK, 3 μM; lane 5). Other samples were treated for 1 hour with biliverdin (50 μM) (lane 6), or biliverdin plus rapamycin (Rapa, 100 nM; lane 7), torin (50 nM ; lane 8) or GSK2334470 (GSK, 3 μM; lane 9).

Supp. Figure 4. *LPS signaling in Raw 264.7 cells.* In (**a**) and (**b**) shown are western blots of Raw 264.7 cells untreated (lane 1) or treated with LPS (100 ng/ml) for the indicated times (lanes 2-6). In (b) a positive control (PC) is shown in lane 6.

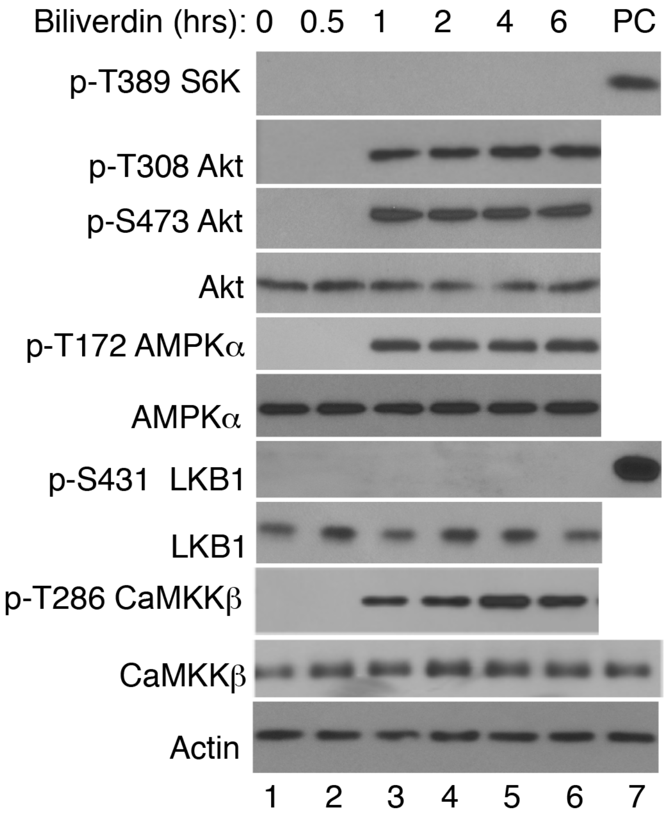

Supplementary Fig. 1

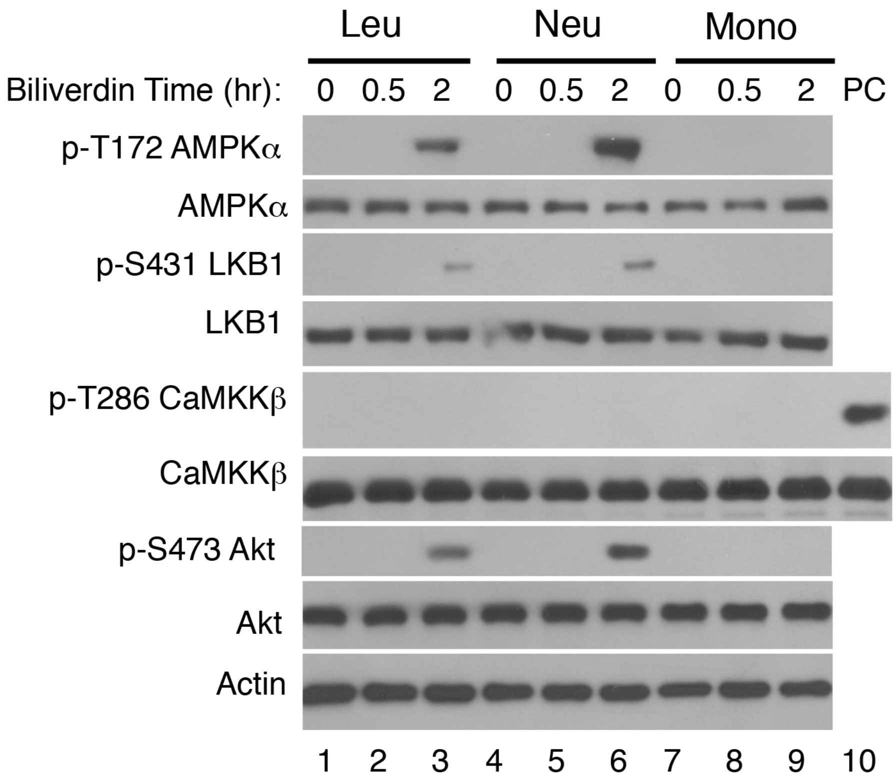

Supplementary Fig. 2

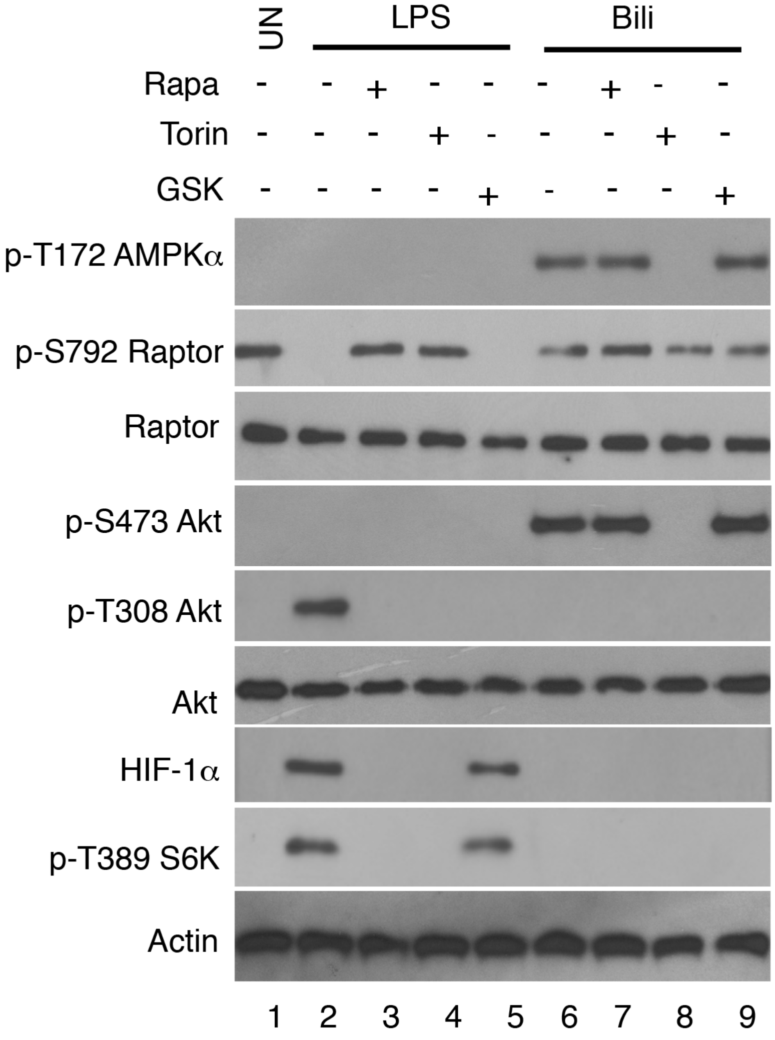

Supplementary Fig. 3

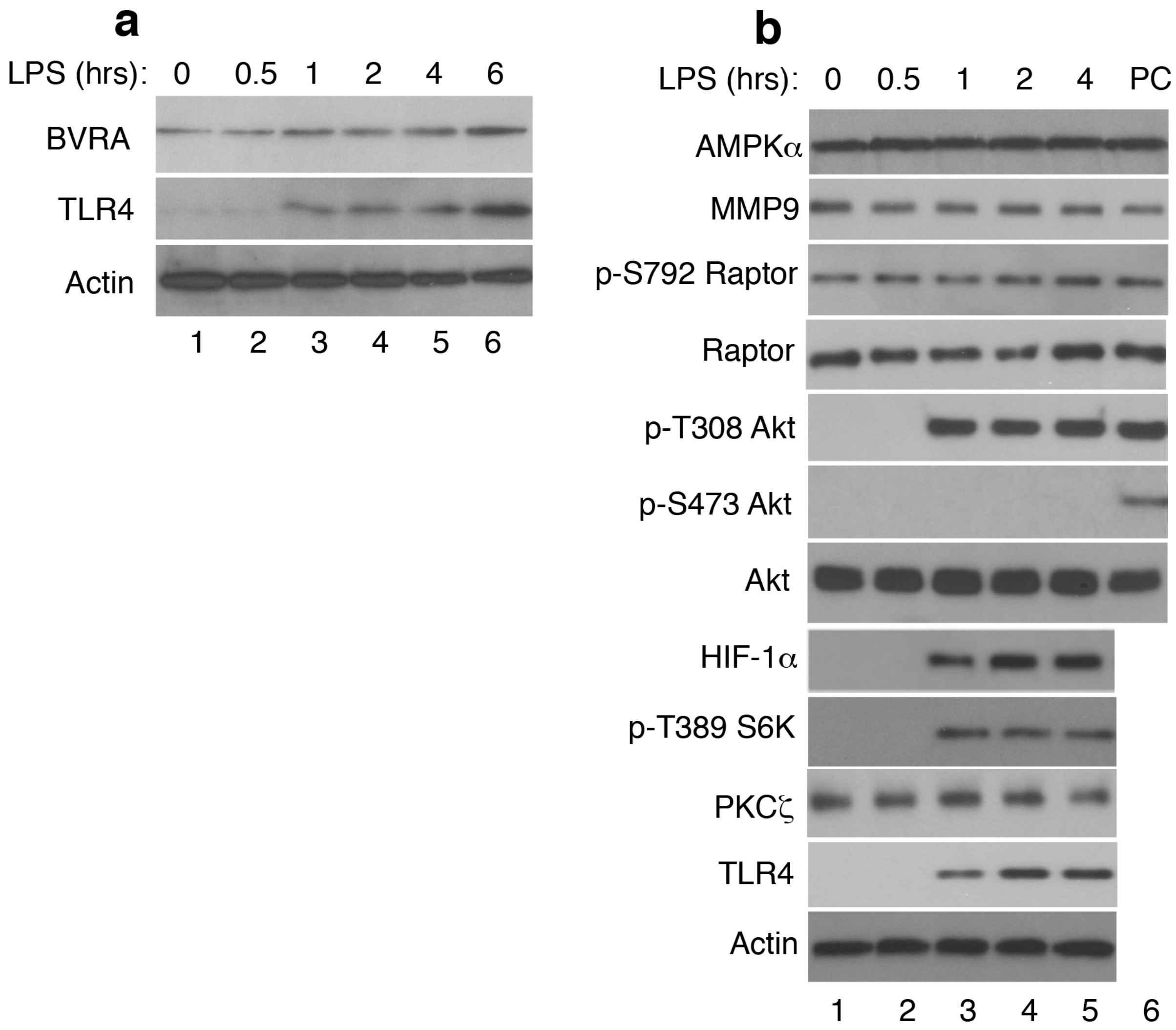

Supplementary Fig. 4
